# MACHINE-LEARNING-BASED INTERPRETABLE PREDICTION FOR MILD COGNITIVE IMPAIRMENT: FROM ADNI DATABASE

**DOI:** 10.1093/geroni/igad104.3707

**Published:** 2023-12-21

**Authors:** Yafei Wu, Maoni Jia, Ya Fang

**Affiliations:** Center for Aging and Health Research, School of Public Health, Xiamen University, Xiamen, China, Xiamen, Fujian, China (People’s Republic); Center for Aging and Health Research, School of Public Health, Xiamen University, Xiamen, China, Xiamen, Fujian, China (People’s Republic); Center for Aging and Health Research, School of Public Health, Xiamen University, Xiamen, China, Xiamen, Fujian, China (People’s Republic)

## Abstract

**Objective:**

An accurate prediction model was still lacking for mild cognitive impairment (MCI). Therefore, this study aimed to predict the 3- and 5-year MCI risk utilizing interpretable machine learning, and to identify possible biomarkers for intervention.

**Methods:**

A total of 444 participants were obtained from the ADNI database. Sociodemographic, neuropsychological, ApoE gene, and structural MRI imaging information was used as predictors. Data was randomly divided into training (70%) and test sets (30%). Six machine learning methods were constructed to predict the risk of MCI. Model performance was assessed by area under the ROC curve (AUC). SHapley Additive exPlanations (SHAP) was used to select important predictors. We also constructed a simplified MCI prediction model for clinical practice.

**Results:**

The mean age (standard deviation) of study population was 73.7 (6.1) years old. A total of 40, and 60 participants progressed into MCI within 3, and 5 years, respectively. Logistic regression (AUC: 0.97) and support vector machine (AUC: 0.93) are the optimal model for 3- and 5-year prediction. The simplified models based on top three neuropsychological test scores also showed high prediction accuracy (AUC > 0.90). APOE gene, intracranial volume, and Clinical Dementia Rating Scale Sum of Boxes (CDRSB) score were top three predictors for 3-year prediction, while CDRSB score, modified Preclinical Alzheimer’s Cognitive Composite Test with Follow-up Test scale (mPACCtrailsB) score, and Functional Activity Questionnaire (FAQ) score were top three predictors for 5-year prediction.

**Conclusion:**

Interpretable machine learning showed promise in early identification of MCI, and would be valuable for targeted prevention.

